# Mutational heterogeneity of angioimmunoblastic T-cell lymphoma indicates distinct lymphomagenic pathways

**DOI:** 10.1038/s41408-017-0047-2

**Published:** 2018-01-17

**Authors:** Mathijs Willemsen, Myrurgia Abdul Hamid, Bjorn Winkens, Axel zur Hausen

**Affiliations:** 1 Department of Pathology, GROW-School for Oncology & Developmental Biology, Maastricht University Medical Centre, Maastricht, The Netherlands; 2 Department of Methodology and Statistics, CAPHRI-Care and Public Health Research Institute, Maastricht University, Maastricht, The Netherlands

The 2016 revision of the World Health Organization classification of lymphoid neoplasms introduces the umbrella category “nodal T-cell lymphomas with T-follicular helper (T_FH_) phenotype”, which includes angioimmunoblastic T-cell lymphoma (AITL), follicular T-cell lymphoma and nodal peripheral T-cell lymphoma (PTCL) with a T_FH_ phenotype^1^. One of the genetic features clustering T_FH_ cell-derived lymphomas is a recurrent *RHOA G17V* mutation, which is present in approximately 60% of investigated cases^2,3^. *RHOA* is a member of the Rho family of GTPases which function as molecular regulators of diverse cellular functions^4^. Mutant RHOA acts as a dominant-negative signaling protein sequestering guanine nucleotide exchange factors (GEFs) thereby inhibiting wildtype RHOA and potentially other GEF-dependent proteins^3^. In vivo, mutant *RHOA* has recently been shown to skew CD4^+^ T-cell differentiation towards the T_FH_ lineage and promote AITL lymphomagenesis^5^. Thus, the *RHOA* *G17V* mutation can be viewed as pivotal genetic aberration in AITL and potentially other T_FH_ cell-derived lymphomas. Mutations contributing to lymphomagenesis in wildtype *RHOA* AITL cases remain largely unknown. This mutational heterogeneity points towards the existence of distinct AITL lymphomagenic pathways. In this report, we explore the mutational landscape of AITL by assessing the data from large sequencing studies focusing on the association between *RHOA* mutational status and recurrent mutations in other genes to provide evidence for the existence of distinct lymphomagenic pathways in AITL.

Sequencing studies of AITL and/or PTCL published between 01-01-2014 and 28-02-2017 using an English language restriction were identified with PubMed. In total, 117 abstracts were screened. Only 34 articles were eligible for full text review. Studies were included in our analysis if they contained ten or more AITL cases and used targeted deep sequencing of *RHOA*, *TET2*, *DNMT3A*, *IDH2*, *CD28* and multiple other genes or whole genome/exome/transcriptome approaches. Also, the original dataset had to be available to the authors. Five of the 34 articles met the prespecified inclusion criteria and were included in our analysis^6–10^. The article selection process was performed by two authors.

In total, these studies analyzed 239 AITL cases using various sequencing techniques. Of interest, in 13.8% (33/239) of investigated AITL cases no detectable mutations were reported. *RHOA* was mutated in 61.1% (146/239) of the investigated AITL cases. The remaining 25.1% (60/239) of cases were wildtype, but carried mutations in other genes (Table 1a).

**Table 1:** *RHOA* mutational status of included studies (A) and association with recurrent mutations in wildtype *RHOA* AITL cases (B)

(A) Author	Sequencing method	AITL cases	No detectable mutations	Wildtype *RHOA*	Mutant *RHOA*^a^
Abate et al.^6^	RNA-seq, TDS (VAV1)	60	15	20	25
Nguyen et al.^7^ 2017	TDS (71 genes)	48	6	9	33
Vallois et al.^9^	TDS (69 genes)	72	8	18	46
Yoo et al.^10^^b^	TDS (70 genes)	29	3	5	21
Palomero et al.^8c^	RNA-seq, TDS (13 genes), allele-specific PCR (*RHOA*)	30	1	8	21
Total		239	33 (13.8%)	60 (25.1%)	146 (61.1%)
**(B) Gene**	**Mutant** *RHOA* AITL cases/total^a^	**Wildtype** *RHOA* AITL cases/total^d^	**Odds ratio**	**95% CI**	*p*-value
*TET2* ^c, e^	101/146 (69.2%)	33/93 (35.5%)	3.46	1.92, 6.22	<0.001
*DNMT3A* ^c, e^	34/146 (23.3%)	10/93 (10.8%)	2.14	0.99, 4.66	0.076
*IDH2* ^c, e^	51/146 (34.9%)	9/93 (9.7%)	6.68	2.89, 15.45	<0.001
*CD28*	14/92 (15.2%)	7/69 (10.1%)	1.73	0.64, 4.73	0.399
*CD28* ^f^	24/92 (26.1%)	8/69 (11.6%)	2.60	0.98, 6.86	0.093
*FYN* ^c^	5/113 (4.4%)	3/78 (3.8%)	1.38	0.30, 6.29	0.972
*PLCG1*	6/92 (6.5%)	6/69 (8.7%)	0.85	0.26, 2.85	0.960
*STAT3*	2/92 (2.2%)	3/69 (4.3%)	0.68	0.13, 3.69	0.979
*VAV1*	2/92 (2.2%)	5/69 (7.2%)	0.27	0.046, 1.56	0.268

*TDS* targeted deep sequencing, *CI* confidence interval

^a^Including non-*G17V* *RHOA* mutations

^b^Only 29/45 AITL cases were analyzed using TDS

^c^Only 30/35 AITL cases were analyzed by allele-specific PCR and targeted deep sequencing. Only one case showed no mutations in both techniques (no detectable mutations). RNA sequencing data not included due to uncertainty of diagnosis

^d^Including cases with no detectable mutations

^e^ Missing data regarding mutational status of *TET2* (*n* = 14), *DNMT3A* (*n* = 14) and *IDH2* (*n* = 10) from Vallois et al. included as not mutated

^f^Including cases with *CTLA4*–*CD28* gene fusion

We focused on the data extract of all wildtype *RHOA* AITL cases to identify potentially recurrent mutations contributing to AITL lymphomagenesis other than *RHOA*. Only mutations occurring in more than 5% of targeted cases and identified in two or more studies were classified as recurrent. *TET2*, *CD28*, *DNTM3A*, *PLCG1*, *IDH2*, *VAV1*, *FYN* and *STAT3* were mutated in 60.7% (34/56), 18.6% (8/43), 17.9% (10/56), 14.0% (6/43), 13.8% (8/58), 11.6% (5/43), 7.8% (4/51) and 7.0% (3/43) of targeted wildtype *RHOA* AITL cases, respectively (Supplementary Data set 1). As these mutations also frequently occur in mutant *RHOA* AITL cases, we performed Mantel–Haenszel statistics to assess the association between these mutations and *RHOA* mutational status across different studies (SPSS v21 IBM Corp., Armonk, NY, USA). A *p*-value ≤0.05 was considered significant.

Statistical analysis showed that mutations in *TET2* and *IDH2* were associated with mutant *RHOA* status (*p* < 0.001 for both genes). Mutations in *DNMT3A* and *CD28*, including *CTLA4*–*CD28* fusion, also tend to show this association (*p* = 0.076 and 0.093). Interestingly, despite being mutated in a low number of cases, mutations in *VAV1* tend to associate with wildtype *RHOA* status (*p* = 0.268). Mutations in *FYN*, *PLCG1* and *STAT3* showed no significant association with *RHOA* mutational status (*p* = 0.972, 0.960 and 0.979) (Table 1b).

This study reports on the association between *RHOA* mutational status and other recurrent mutations in AITL. We found an association between mutant *RHOA* and mutations in *TET2* and *IDH2*. Despite being mutually exclusive in acute myeloid leukemia, mutations in *IDH2* and *TET2* tend to co-occur in *AITL*^11^. Gene expression profiling and promoter methylation analysis of double mutant AITL cases showed upregulation of genes associated with T_FH_ phenotype and downregulation of genes associated with T_H_1 phenotype^11^. Mutant *IDH2* and *TET2* potentially cooperate with mutant *RHOA* to induce a potent T_FH_ phenotype in vivo. This mechanism would explain the association found between these mutations in the present study. We also identified a strong tendency towards association between mutant *RHOA* and mutations in *DNMT3A*. The exact mechanism by which mutations in epigenetic modifiers contribute to lymphomagenesis remain to be elucidated, but alterations in hematopoietic stem cell differentiation is an attractive theory. The present study also identified a strong tendency towards association between mutant *RHOA* and mutations in *CD28*, including *CTLA4*–*CD28* gene fusion. *CD28* mutations in AITL are confined to hotspot residues D124 and T195 and render CD28 constitutively active^10,12^. The *CTLA4*–*CD28* fusion gene has only been reported in an Asian cohort^10^. Therefore, validation of this fusion gene in other cohorts is essential to confirm the association between mutant *RHOA* and mutations in *CD28*. Altogether, these findings point towards a classic AITL lymphomagenic pathway (Fig. 1). Several therapeutic approaches targeting epigenetic modifiers, IDH2 or CD28 are currently in clinical trials or have already been approved for other diseases^13,14^. The tendency of these mutations to cluster will potentially help to develop novel combinatorial therapeutic regimens.

**Fig. 1:**
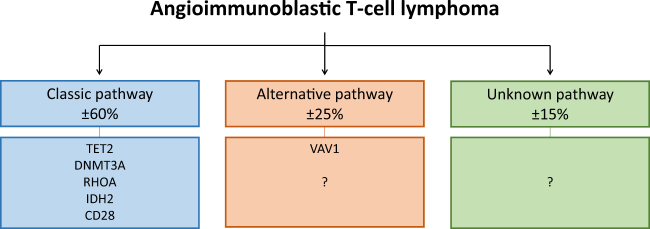
Three distinct lymphomagenic pathways in AITL. Our statistical analysis has identified three potentially distinct lymphomagenic pathways in AITL. The classic pathway accounts for approximately 60% of AITL cases and is characterized by the *RHOA* *G17V* mutation in association with several other recurrent mutations. The alternative pathway accounts for approximately 25% of AITL cases and is characterized by mutations in *VAV1* or potentially other members of the Rho family of GTPases or their regulatory proteins. The mutations that drive the remaining approximately 15% of AITL cases are unknown. It is possible that mutations in signaling pathways directly regulating T_FH_ differentiation contribute to these cases

Despite being mutated in rather a low number of AITL cases, this study identified the tendency of mutations in *VAV1* to associate with wildtype *RHOA*. *VAV1* encodes a Rho GTPase family-specific GEF which is primarily expressed in the hematopoietic system^15^. The studies that targeted *VAV1* identified three missense mutations (*E524D*, *E556D* and *D797G*), two frameshift deletions (*151_158del* and *778_783del*), one fusion gene (*VAV1*–*S100A7*) and one in-frame deletion (*778_786del*)^6,9,10^. Abate et al. found the *778_786* in-frame deletion and *VAV1*–*S100A7* fusion gene to be locked in a constitutively active conformation, indicated by high levels of Tyr174 phosphorylation^6^. Both genetic aberrations resulted in increased VAV1 catalytic-dependent functions downstream of RAC1, another member of the Rho family of GTPases^6^. These findings are in accordance with previous experiments showing that constitutively active VAV1 predominantly increases nucleotide exchange of RAC1 and to a lesser extend of RHOA^16^. Interestingly, the RAC1 pathway is upregulated in mutant *RHOA* compared to wildtype *RHOA* AITL cases, providing evidence that both mutations have similar effects on VAV1 catalytic-dependent pathways^17^. Additionally, Abate et al. found that the *VAV1*–*S100A7* fusion gene resulted in increased NFAT activity, a functional readout of VAV1 non-catalytic activity, whereas both the *778_786* in-frame deletion and *VAV1*–*S100A7* fusion gene increased expression of NFAT target genes^6^. A recently published study, not yet indexed by PubMed at the time of our search, identified activating *VAV1* mutations in 8.2% (7/85) of wildtype *RHOA* AITL cases, compared to 0% (0/41) in mutant *RHOA* AITL cases, respectively^18^. They also showed that mutant RHOA enhances the non-catalytic functions of VAV1 through increased Tyr174 phosphorylation, thereby increasing NFAT activity and expression of NFAT target genes. Together, these data not only strengthen the association between mutant *VAV1* and wildtype *RHOA*, but also provide evidence that mutant *RHOA* and mutant *VAV1* have similar effects on catalytic and non-catalytic signaling pathways downstream of VAV1. Therefore, we deduce from these data that mutant *RHOA* and mutant *VAV1* contribute to AITL lymphomagenesis in a similar manner. This would mean that *VAV1* is part of an alternative AITL lymphomagenic pathway (Fig. 1). Previous clinicopathological studies have shown that mutant *RHOA* AITL cases have worse performance status, more frequent B-symptoms and splenomegaly and a more potent T_FH_ immunophenotype compared to wildtype *RHOA* AITL cases^19,20^. These data provide additional justification for separating AITL subgroups.

According to our analysis, no mutations were detectable in approximately 15% (range 3–25%) of AITL cases (Fig. 1). Exploring the mutational landscape of AITL using targeted deep sequencing panels enriched with members of the Rho family of GTPases and their regulatory proteins might identify driver mutations in this subgroup. It is also possible that other lymphomagenic mechanisms contribute to some AITL cases, for example mutations in signaling pathways directly regulating T_FH_ differentiation.

We are aware that there are some limitations to our study. Our findings are entirely based on retrospective data from a relatively small sample size. Furthermore, there is significant technical heterogeneity between the sequencing studies from which the data is derived. The individual studies use different sequencing techniques, bioinformatics pipelines for data processing and mutation calling methods. Despite these limitations, this study remains noteworthy as it provides a unique perspective on associations and possible collaborations between the most common genetic aberrations in AITL as well as providing a rationale for future research.

In short, using data from large sequencing studies this study reports on varying associations between *RHOA* mutational status and other recurrent mutations in AITL. These findings enable us to identify three potentially distinct AITL lymphomagenic pathways. First, the classic pathway with the *RHOA* *G17V* mutation which is associated with mutations in *TET2*, *DNMT3A*, *IDH2* and *CD28*. Secondly, the alternative pathway with mutations in *VAV1* or potentially yet unidentified mutations in members of the Rho family of GTPases or their regulatory proteins. Third, AITL cases with unknown mutations which might arise from direct mutations in pathways regulating T_FH_ differentiation. To what extend these different lymhpomagenic pathways result in different clinical behavior of AITL is largely unknown. Additional evidence on the mutational landscape of AITL, especially wildtype *RHOA* AITL cases, is needed to either confirm or refute our findings. Furthermore, prospective data is needed to identify potential clinical differences between the distinct lymphomagenic pathways of AITL proposed in this manuscript.

## Electronic supplementary material

Supplementary dataset 1
